# How collagen becomes ‘stiff’

**DOI:** 10.7554/eLife.77041

**Published:** 2022-02-21

**Authors:** Rebecca G Wells

**Affiliations:** 1 Department of Medicine, University of Pennsylvania Philadelphia United States

**Keywords:** fibrosis, collagen, lung, Human

## Abstract

Oxidative stress following a lung injury can alter the structure of collagen, causing it to stiffen and trigger the formation of a fibrotic scar that further hardens the tissue.

**Related research article** Brereton C, Yao L, Zhou Y, Vukmirovic M, Bell J, Ridley RA, Davies ER, Dean LSN, Andriotis OG, Conforti F, Mohammed S, Wallis T, Tavassoli A, Ewing R, Alzetani A, Marshall BG, Fletcher SV, Thurner PJ, Fabre A, Kaminski N, Richeldi L, Bhaskar A, Loxham M, Davies DE, Wang Y, Jones MG. 2022. Pseudohypoxic HIF pathway activation dysregulates collagen structure-function in human lung fibrosis. *eLife* **11**:e69348. doi: 10.7554/eLife.69348.

When our bodies heal following an injury, fibroblasts and other cells deposit components of the extracellular matrix, such as collagen, that form a ‘scar’ over the damaged area. This process, known as fibrosis, leads to a build-up of stiff fibrotic tissue that interferes with the activity of the underlying organ.

In healthy organs, fibroblasts lay down just enough collagen to maintain the integrity of the tissue (D’Urso and Kurniawan, 2020). However, if the tissue begins to stiffen, these cells produce more collagen, disrupting this equilibrium (Tschumperlin et al., 2018). This increases the rigidity of the organ, triggering the fibroblasts to release even more collagen. While this explains how fibrosis progresses, it is less clear how the cycle starts.

Previous studies showed that fibrosis is initiated by changes in the mechanical properties of collagen that arise from modifications to its structure (Jones et al., 2018; Georges et al., 2007). The experiments found that, after a tissue injury, a family of enzymes called lysyl oxidases (or LOXs for short) increase the bonds between individual collagen fibers in the organ. This modified structure makes the tissue stiffer, driving the fibrotic process. Now, in eLife, Mark Jones from the University of Southampton and co-workers – including Christopher Brereton and Liudi Yao as joint first authors – report on the pathway that leads to this altered collagen architecture in patients with pulmonary fibrosis (Brereton et al., 2022).

First, the team (who are based at various institutes in the United Kingdom, the United States, Austria, Italy and Ireland) studied lung tissue from individuals with idiopathic pulmonary fibrosis, a disease of unknown cause in which patient’s lungs are severely scarred (Richeldi et al., 2017). They found that the genes coding for two enzymes that sequentially modify collagen (Yamauchi and Sricholpech, 2012) – lysyl hydroxylase PLOD2 and LOXL2 – were highly expressed in the same lung cells at the same time. The gene for the most commonly found type of collagen in fibrosis (called type I) was also activated in the lung tissue, but its pattern of expression did not correlate with the genes for PLOD2 or LOXL2. This suggests that it is regulated independently from these two enzymes.

Next, Brereton, Yao et al. extracted fibroblasts from patients with idiopathic pulmonary fibrosis and cultured them in the laboratory for six weeks, treating the cells with several growth factors associated with fibrosis. The experiments showed that a family of proteins called Hypoxia Inducible Factors (HIFs), which regulate the body’s response to varying oxygen levels, activated the genes for PLOD2 and LOXL2. This increased the number of cross-links between the collagen fibers and made the fibers stiffer. Imaging the collagen produced by these cells with an electron microscope revealed that the fibers had a smaller diameter, as had previously been observed in patients with idiopathic pulmonary fibrosis (Jones et al., 2018). This suggests that HIFs regulate tissue mechanics not by increasing the amount of collagen, but by altering collagen’s structure.

HIF is negatively regulated by another protein called FIH (short for Factor Inhibiting HIF), which is inactivated by the unstable byproducts of oxygen metabolism, also known as oxidative stress (Lando et al., 2002). Loss of FIH causes cells to enter ‘pseudohypoxia’, a state in which cells behave as if oxygen levels are low despite being in normal conditions (Masson et al., 2012). Brereton, Yao et al. found that reducing FIH – both in cells cultured in the laboratory as well as in lung tissue from patients with idiopathic pulmonary fibrosis – led to higher levels of HIF, which increased the activity of PLOD2 and LOX2, causing changes to collagen’s architecture and fibrosis.

These findings suggest that the pseudohypoxia state induced by oxidative stress is central to the pathology of idiopathic pulmonary fibrosis, and may help to explain how the fibrosis cycle starts (Figure 1): injuring or damaging the lung leads to a rise in oxidative stress, which dampens the level of FIH. This allows HIF to activate the genes for the enzymes PLOD2 and LOXL2, which then sequentially alter the structure of collagen. These changes increase the stiffness of the tissue, initiating the fibrosis cycle.

**Figure 1.**
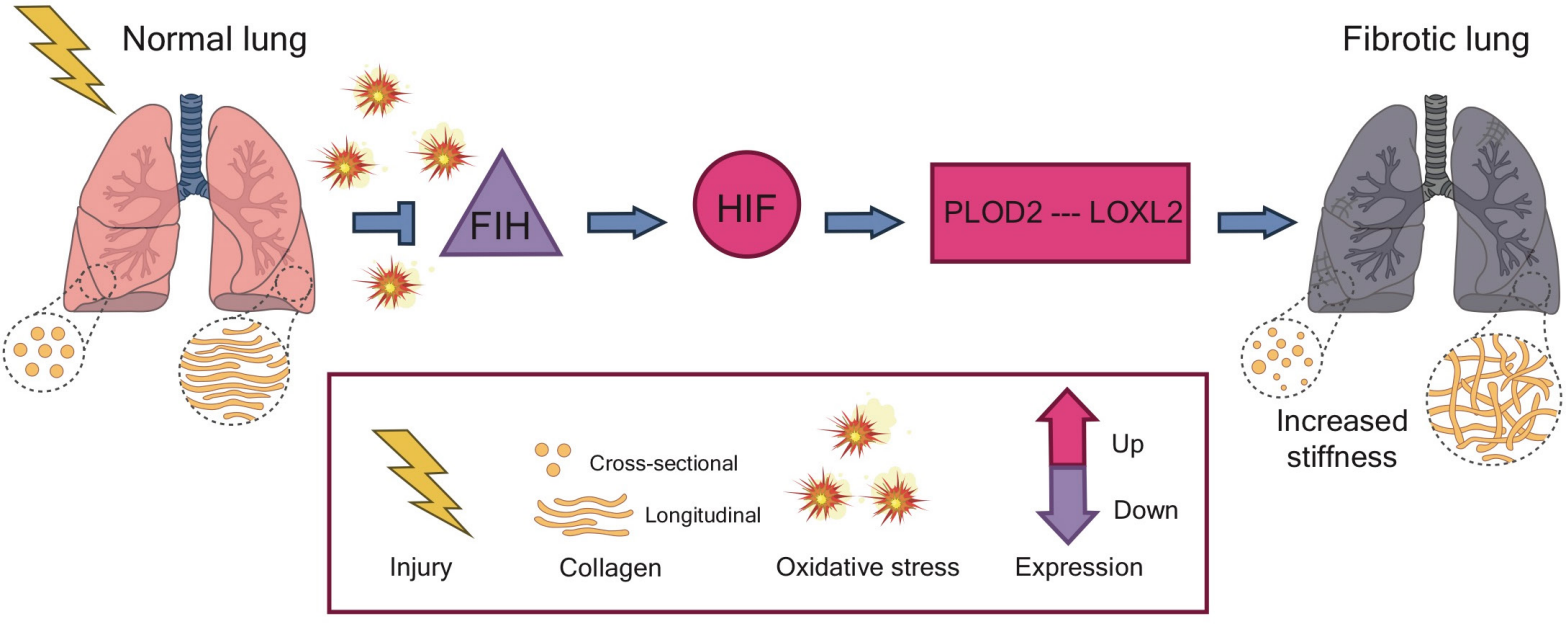
The pathway to fibrosis. When cells in the lung become injured or damaged (left), the resulting increase in oxidative stress leads to a decrease in the expression of the genes that code for a protein called FIH (purple triangle). This results in a pseudohypoxia state which increases the activity of the protein HIF (pink circle), allowing it to upregulate the expression of the genes that code for two enzymes: PLOD2 and LOXL2. These enzymes act in sequence to increase and strengthen the bonds, or ‘cross-links’, between fibers of collagen. The altered structure stiffens the tissue, triggering cells in the lung to deposit more collagen and start the process of fibrosis. HIF: Hypoxia Inducible Factor; FIH: Factor Inhibiting HIF.

Image credit: Iris de Jong (CC BY 4.0).

This work provides new insights in to how fibrosis is triggered. Particularly intriguing is the suggestion that intervening at the level FIH or HIF may be better at treating idiopathic pulmonary fibrosis than targeting the machinery that synthesizes collagen, which is currently considered to be the most effective approach. Further work is needed to fully understand how the architecture of collagen becomes dysregulated and to test how substances that block this newly discovered pathway impact fibrosis.
